# The Effects of TRPC6 Knockout in Animal Models of Kidney Disease

**DOI:** 10.3390/biom12111710

**Published:** 2022-11-18

**Authors:** Stuart E. Dryer, Eun Young Kim

**Affiliations:** 1 Department of Biology and Biochemistry, University of Houston, Houston, TX 77204-5001, USA; 2 Department of Biomedical Sciences, Tilman J. Fertitta Family College of Medicine, University of Houston, Houston, TX 77204-5001, USA

**Keywords:** TRPC6, podocyte, mesangial cells, glomerulosclerosis, renal fibrosis, diabetic nephropathy

## Abstract

Diseases that induce a loss of renal function affect a substantial portion of the world’s population and can range from a slight decline in the glomerular filtration rate or microalbuminuria to complete kidney failure. Kidney disorders can be acute or chronic, but any significant reduction in renal function is associated with increased all-cause morbidity and mortality, especially when the conditions become chronic. There is an urgent need for new therapeutic approaches to slow or halt the progression of kidney disease. One potential target of considerable interest is the canonical transient receptor potential-6 (TRPC6) channel. TRCP6 is a cationic channel with a significant permeability to Ca^2+^. It is expressed in several tissues, including in multiple cell types of the kidney in glomeruli, microvasculature, and tubules. Here, we will describe TRPC6 channels and their roles in signal transduction, with an emphasis on renal cells, and the studies implicating TRPC6 channels in the progression of inherited and acquired kidney diseases. We then describe studies using TRPC6 knockout mice and rats subjected to treatments that model human diseases, including nephrotic syndromes, diabetic nephropathy, autoimmune glomerulonephritis, and acute kidney injuries induced by renal ischemia and by obstruction of the urinary tract. TRPC6 knockout has been shown to reduce glomerular manifestations of disease in several of these models and reduces renal fibrosis caused by urinary tract obstruction. TRPC6 knockout has proven to be less effective at reducing diabetic nephropathy in mouse and rat models. We also summarize the implications of these studies for drug development.

## 1. Introduction

It has been estimated that up to 14% of the adult population in the United States suffers from a significant loss of renal function based on reduced estimated glomerular filtration rate or albuminuria [1]. Many cases of chronic kidney disease (CKD) are due to pathological processes that at least initially impact renal glomeruli, and glomerular diseases remain the most common cause of end-stage kidney failure (ESKF). Examples of potentially devastating glomerular diseases include diabetic nephropathy, various forms of autoimmune glomerulonephritis, and various primary nephrotic syndromes [1]. However, in recent years there have been increases in the incidence of acute kidney injuries (AKI) that are typically associated with insults to renal tubules, and especially to the proximal tubule [2]. AKI usually occurs secondary to other conditions, for example, following urinary tract obstruction or due to renal ischemia following shock, cardiac arrest, or following certain surgical procedures [2]. It is also a common complication in patients undergoing intensive care for COVID-19 [3]. There is a current unmet need for pharmacotherapies that can slow or halt the progression of various forms of CKD, as well as the deleterious renal responses to AKI.

Ion channels have historically proved to be useful drug targets and it has been estimated that ~15% of all currently used drugs target ion channels [4]. Some, such as calcium channel blockers, are in very widespread clinical use. Ion channels are attractive drug targets because many of them are accessibly located on the cell surface, because they are particularly susceptible to inhibition or modulation by small molecules with drug-like properties and because they are amenable to high-throughput drug discovery strategies. Nevertheless, prior to undertaking a costly drug discovery effort, it is useful to have information from knockout or transgenic animals designed to assess whether inhibition or modulation of a particular channel would be useful in a particular disease state and to assess whether obvious toxicities can be expected in the usual case when a particular channel is expressed in multiple tissues. Human genetic evidence that implicates an ion channel in a particular disease process can also be useful in identifying drug targets.

One ion channel that has received particular attention in the context of kidney disease is the canonical transient receptor potential-6 channel (TRPC6) [5,6]. The interest in TRPC6 as a therapeutic target is based on evidence from both humans and animals. The purpose of this review is to summarize the evidence to date on the potential of TRPC6 as a drug target, with a particular emphasis on the effects of TRPC6 knockouts in animal models of kidney disease.

## 2. Properties of TRPC6 Channels

TRPC6 is a widely expressed cation channel that plays multiple roles in signal transduction in different organs [5,6]. Functional TRPC6 channels are tetrameric proteins. Individual TRPC6 subunits have six transmembrane alpha-helical domains, and the cytosolic portions of the amino and carboxyl terminals are quite large and contain several regulatory motifs and protein-interaction domains [5,6]. TRPC6 channels are permeable to Na^+^, K^+^, and Ca^2+^ [7,8] as well as to certain larger metal ions [9], and therefore the activation of these channels on the cell surface will always result in cell depolarization. In excitable cells, activation of TRPC6 channels through G protein-coupled receptors (GPCRs) leads to activation of voltage-activated Ca^2+^ channels (Ca*_v_*), leading to Ca^2+^ influx and changes in cell motility or secretion. In those cellular contexts, TRPC6 typically functions as a receptor-operated cation channel [10], and its permeability to monovalent cations is sufficient to initiate downstream physiological events. However, in non-excitable cells that do not typically express functional Ca*_v_* channels, the TRPC6 channels are themselves a physiologically significant source of Ca^2+^. Because the permeability of TRPC6 to Ca^2+^ is relatively limited and is markedly reduced as a result of membrane depolarization [7], TRPC6 channels in non-excitable cells must co-localize closely with their downstream effector proteins. As such, they are often components of larger molecular signaling complexes that can include other channels and transporters [5,6].

TRPC6 is a member of the family of canonical transient receptor potential (TRPC) channels and is most closely related to TRPC3 and TRPC7. It can form functional heteromultimers with TRPC3 and TRPC7 but is not able to form heteromultimers with either TRPC4 or TRPC5 [10]. There are reports that TRPC6-TRPC3 heteromers have slightly different properties than either of the channels expressed by themselves [11]. It is quite common for TRPC6 and TRPC3 to be co-expressed in many cell types, including in the kidney [12], and therefore one must consider that at least some of the TRPC6 channels in situ may be heterotetrameric.

As with other TRP channels, TRPC6 channels can become active in response to biophysically distinct stimuli; in other words, their gating at least at the cellular level appears to be multimodal. The most extensively studied mode is via activation of GPCRs that are coupled via the G protein G_q_ to activation of phospholipase C. This pathway leads to generation of diacylglycerol (DAG), which directly activate the channels [10,13], probably by binding to domains that stabilize the open state [14,15]. In podocytes, DAG can also drive signaling cascades that lead to generation of reactive oxygen species (ROS) in the immediate vicinity of TRPC6 channels, leading to their rapid exocytotic insertion into the plasma membrane and to their activation [16]. In this regard, TRPC6 channels can move into the plasma membrane within seconds after the activation of GPCRs [17]. In podocytes this occurs in response to angiotensin II acting on AT1 receptors [18,19] or ATP acting on P2Y receptors [20], and TRPC6 activation by those pathways is completely blocked when ROS are quenched [18,19,20].

**Figure 1:**
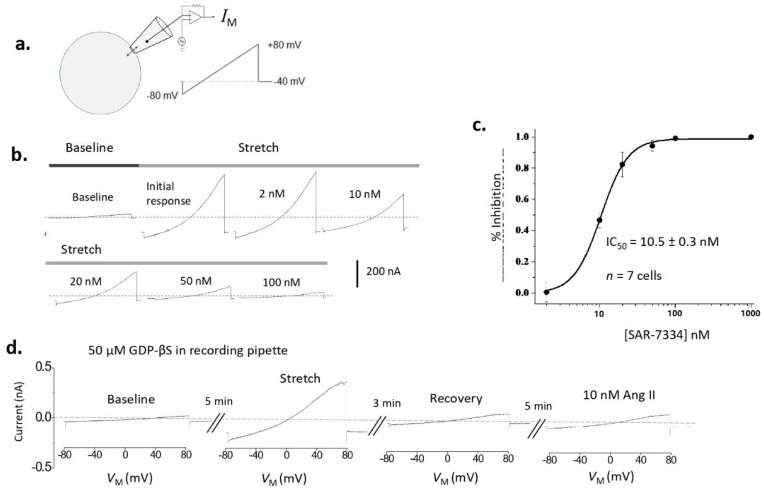
Activation of TRPC6 channels in podocytes by hypoosmotic stretch of the plasma membrane. Membrane stretch was evoked by superfusion of an extracellular saline that is 70% as concentrated as that used when whole-cell contact is initiated (Baseline). (**a**) Whole-cell recording configuration and ramp voltage command to characterize cationic currents. Details of recording conditions, including the voltage-clamp protocol, and the composition of pipette and extracellular solutions are described in [23]. (**b**) Cationic currents evoked by membrane stretch are progressively blocked by increasing concentrations of the selective TRPC6 inhibitor SAR-7334. This recording was made from podocytes in an isolated rat glomerulus preparation [5]. (**c**) Dose-response curve for SAR-7334 in the types of experiments shown in (**b**). (**d**) Stretch-evoked TRPC6 current in a cultured podocyte evoked with a recording pipette containing 50 μM GDP-βS, which will block all G protein-mediated signaling in the cell. Stretch-evoked currents are reversible in podocytes and subsequent exposure to 10 nM angiotensin II fails to evoke a response. Modified from [23].

TRPC6 channels in cells can also become active in response to mechanical stimuli and this was initially shown to be necessary for autoregulation of cerebral arteries [21]. While it has been argued that this reflects an intrinsic mechanosensitivity of certain GPCRs [22], in podocytes the process whereby TRPC6 channels are activated by membrane stretch is biophysically and pharmacologically distinct from activation mediated by GPCRs (Figure 1). Thus, mechanical activation of TRPC6 in podocytes persists when G protein signaling is completely blocked by including GDP-βS in the recording pipette, it is inhibited by toxins thought to selectively target mechanosensitive channels, it persists in the presence of ROS quenchers that completely block TRPC6 activation by GPCRs, and it can be enhanced by knocking down interacting proteins such as podocin that are required for TRPC6 activation by GPCRs [23]. Moreover, mechanical activation of TRPC6 in heterologous expression systems is eliminated by a mutation in TRPC6 (TRPC6-N143S) that leads to slightly enhanced activation by GPCR signaling [24]. We should note, however, that both mechanical and GPCR activation of podocyte TRPC6 channels are completely blocked by agents such as SAR-7334 and La^3+^ that act directly on the channels [5]. Mechanical activation of TRPC6 in podocytes is enhanced following disruption of actin filaments with cytochalasin D, raising the possibility that this mode of activation is regulated by some sort of interaction with the cytoskeleton [23]. Mechanical activation of TRPC6 channels has also been observed in glomerular cells in response to increases in intraglomerular pressure in vivo [25], which could reflect both direct mechanical activation of TRPC6 and activation via paracrine purinergic signaling. It has been argued that enhanced mechanical activation of TRPC6 in certain kidney disease states may be a factor that contributes to renal pathology [5,6,23], as will be discussed further below.

## 3. TRPC6 Expression in the Mammalian Kidney

Attempts to map the distribution of TRPC6 channels in the kidney have been limited by the underlying cell biology of these proteins and by the methodologies used. An important caveat to all those studies is that an ion channel can be physiologically important in a cell in which it is expressed at very low levels. Activation of a very small number of cation channels can lead to a substantial depolarization depending on whether other channels are active at the same time, and the influx of a relatively small number of Ca^2+^ ions can lead to robust activation of downstream signaling proteins if they are co-localized within a few tens of nanometers of the channel pore [26]. It should also be noted that many of the commercial TRPC6 antibodies have questionable selectivity (which can vary considerably from one lot to the next), and therefore immunohistochemical and immunofluorescence studies of TRPC6 distribution need to be interpreted with caution.

With those caveats, we note that immunofluorescence analysis of mouse kidney carried out with extensively purified and characterized antibodies revealed TRPC6 and TRPC3 expression in glomerular cells, notably mesangial cells and podocytes, and in principal cells of the collecting duct, where they co-localize with aquaporin-2 [6,12]. Consistent with that, studies on polarized epithelial cell lines derived from the collecting duct detected TRPC3 on the apical surface whereas TRPC6 was present on both the apical and basolateral surface [12]. TRPC6 channels are also present in macrophages, fibroblasts and dendritic cells that are committed to immune activation and that are present in the renal interstitium [27], and there is evidence that they are also present in afferent or efferent arterioles of glomeruli [28]. However, among the various renal cells, the most detailed functional studies of TRPC6 using single-channel and whole-cell recording and Ca^2+^ imaging techniques have been carried out in mesangial cells and in podocytes [6].

TRPC6 channels in podocytes are expressed in the slit diaphragm domains of foot processes as well as in the cell body [29,30]. They appear to regulate different biochemical processes in those compartments, e.g., changes in cytoskeletal dynamics in foot processes, and regulation of transcription in the cell body [5,6,31,32,33]. There is often an increase in TRPC6 abundance in human kidney diseases and in kidney disease models in mice and rats, and in podocytes there are disease conditions that induce a marked increase in TRPC6 currents and altered gating properties [5]. It has also been shown that activation of TRPC6 can lead to an increase in its own transcription in podocytes [32], and TRPC6 can therefore function as part of a positive feedback pathway that drives the progression of kidney disease. TRPC6 channels in mesangial cells may contribute to contraction [33] and proliferation [34] of these cells, especially in response to factors such as angiotensin II [34]. In subsequent sections, we will describe changes in TRPC6 that have been observed in human diseases and in animal disease models, and we will then review studies that test the hypothesis that knockout or inactivation of TRPC6 will slow the progression of kidney disease.

## 4. TRPC6 Channels in Glomerular Disease

In 2005 a pair of landmark papers showed that mutations in *TRPC6*, the gene encoding TRPC6 channels, can cause severe familial forms of focal and segmental glomerulosclerosis, with the initial presentation of disease usually occurring in the second or third decade of life, and often progressing to ESKF [29,35]. Most of these mutations cause a marked gain-of-function when the channels are expressed in heterologous expression systems and activated by co-expressed GPCRs. The mechanisms whereby these mutations cause a gain-of-function probably depend on which mutation is considered. For example, TRPC6-P112Q, the first one discovered, is likely to be a trafficking mutant that leads to abnormally increased steady-state abundance of TRPC6 subunits on cell surface [35]. Several others, such as TRPC6-M132T, may enhance gating by de-stabilizing the closed state of the channel [14,15]. Since those initial reports, many other TRPC6 mutations have been identified in patients with nephrotic syndromes [36,37,38,39,40,41,42,43]. The disease-associated TRPC6 mutations described to date are found in residues that are conserved in other TRPC family proteins, specifically in the ankyrin-repeat domains near the amino terminus or in other cytosolic domains near the carboxyl terminal. A recent structural study has shown the existence of inhibitory and activating Ca^2+^-binding sites in TRPC3 that are conserved in TRPC6 [15]. These sites couple intracellular Ca^2+^ concentrations to basal channel activity. Moreover, gain-of-function mutations of TRPC6 such as M132T appear to activate the channel by allosterically abolishing the inhibitory effects of intracellular Ca^2+^ [15]. To date, no disease-associated TRPC6 mutations have been detected in transmembrane alpha helices or in the putative pore domains. A small number of disease-associated mutations, such as TRPC6-G757D, have been found to result in a loss of function, and indeed seem to function in a dominant-negative manner, at least when channels are activated by GPCRs [44]. One would therefore expect those mutations to cause a loss of function if they were to heteromerize with TRPC3 and one might observe reduced channel activity in heterozygous individuals.

Collectively, these data suggest that some amount of TRPC6 (and/or TRPC3) activity contributes to the formation of normal glomeruli or their subsequent function but that sustained hyperactivation of TRPC6 also leads to glomerular disease. Consistent with this, podocyte-specific expression of disease-causing TRPC6 mutations, especially TRPC6-M131T, results in albuminuria and glomerulosclerosis [45] albeit less than occurs in humans with a homologous mutation. Thus, TRPC6-M131T is the mouse homolog of human TRPC6-M132T, which was discovered in an individual with severe FSGS with a childhood onset and which produces a very large gain-of-function in heterologous expression systems [36]. The available studies at least suggest that gain-of-function TRPC6 mutations that produce later onset nephrosis in humans tend to produce less severe kidney disease in mice [45,46,47]. Familial nephrotic syndromes are quite rare, and only a small proportion of the familial forms are due to mutations in *TRPC6*. Indeed, familial nephrotic syndromes are far more likely to be caused by mutations in *NPHS2*, the gene that encodes podocin [48], a protein that among other things regulates TRPC6 gating in podocytes [23].

Acquired glomerular diseases, including various glomerulonephritides and nephrotic syndromes, are far more common. Therefore, it is notable that wild-type TRPC6 channels may be dysregulated in acquired forms of glomerular disease. An early study documented an increase in the expression of TRPC6 transcripts and proteins in glomeruli of patients with minimal change disease, membranous glomerulonephritis, and to a lesser extent in patients with primary FSGS [49]. This has also been observed in rat models of glomerular disease, for example in acute and chronic puromycin aminonucleoside (PAN) nephrosis [50], autoimmune glomerulonephritis [49,51] and in aging [52]. Changes in overall abundance do not necessarily imply a change in function because channels are often regulated by trafficking or by covalent modification, e.g., phosphorylation. However, functional changes in glomerular TRPC6 channels have been observed in rats during chronic PAN nephrosis [5]. Thus, whole-cell recordings from podocytes in glomeruli acutely isolated during chronic PAN nephrosis exhibited an almost order of magnitude increase in TRPC6 current evoked by membrane stretch compared to currents recorded from vehicle-treated healthy controls. These stretch-evoked currents were blocked by the selective TRPC6 inhibitor SAR-7334 [5]. In addition, currents evoked through GPCRs were reduced in the chronic PAN model, which probably reflects a marked decrease in the abundance of podocin [5]. A very similar pattern is observed in cultured podocytes exposed to serum or plasma samples of patients with recurrent forms of primary FSGS compared to samples from healthy people [53,54]. This effect is mimicked by exposing cultured podocytes to recombinant preparations of the soluble urokinase receptor (suPAR) [53,54], one of several factors implicated in the pathogenesis of primary FSGS [55]. Given that podocytes are always in a mechanically dynamic environment [56] and that gating of podocyte TRPC6 channels occurs in response to changes in intraglomerular pressure [25], these gating changes would be expected to result in increases in Ca^2+^ influx over a sustained period.

## 5. Effects of TRPC6 Knockout in Animal Models of Kidney Disease

Studies such as those described above suggest that sustained excessive activation of TRPC6 channels can drive the progression of glomerular disease, at least in part through Ca^2+^ overload [31,32,57], although other mechanisms may contribute [58]. At least part of the deleterious effects of TRPC6 activation are probably due to activation of calcineurin-NFATc cascades [30,31,57], which would be expected to cause substantial changes in gene expression in podocytes and elsewhere [30,31]. Notably, this includes increases in the transcription of *TRPC6*, and thus TRPC6 activation over time will lead to increases in its own biosynthesis [31]. Sustained Ca^2+^ overload in principle can activate a host of downstream transduction mechanisms that in cultured cells leads to apoptotic cell death [57]. TRPC6 activation also leads to activation of calpain, which by analogy to neurodegenerative states can produce a variety of downstream changes in cytoskeletal arrangements and the ability of cells to adhere to a substrate [58]. It is interesting that the activation of calpain may be Ca^2+^-independent, and may depend on some type of conformational coupling that requires a direct between TRPC6 and calpain [58]. In any case, manipulations that reduce the number of active TRPC6 channels would be expected to attenuate glomerular pathologies. Several studies to test this idea have made use of TRPC6 knockout models in mice and rats and have examined models of acquired forms of glomerular disease. These studies are briefly outlined in Table 1. In the rest of this section, we will summarize results obtained in these studies in more detail.

### 5.1. Puromycin Aminonucleoside (PAN) Nephrosis

One widely used model of an acquired glomerular disease is the chronic PAN nephrosis model mentioned earlier. In this model, PAN is administered to rats typically by one or more intraperitoneal injections [69,70]. PAN appears to be selectively taken up by podocytes and its subsequent metabolism leads to a massive generation of ROS. Over a period of days this causes the rapid death of a certain fraction of podocytes due to oxidative effects throughout the cells. Shortly following the initial PAN injection there is a large increase in urine albumin excretion, which in rats typically peaks at 9–14 days [70]. These time points are referred to as the acute phase of the PAN nephrosis model. It is caused by the acute toxic effects of the drug on podocytes, and while it is sometimes discussed as a model for minimal change nephrotic syndrome, it is not a direct analog to any human disease. During this acute phase in rats the glomeruli appear mostly normal by light-level microscopy, but effacement of foot processes can be seen with electron microscopy. After that acute phase, the animals appear to recover based on measurements of urine albumin excretion. However, if the percentage of podocytes killed by the initial PAN insult exceeds a threshold of approximately 25% the albuminuria will once again begin to rise, and by 30 days after the initial injection it is again possible to detect nephrotic range albuminuria, which is now accompanied by glomerulosclerosis readily observed at the light microscopic level [70]. The glomerulosclerosis and fibrosis become much more extensive if a second PAN injection is given. Chronic PAN nephrosis is considered a model for adaptive forms of FSGS [70], a common pathological observation in patients with severe uncontrolled hypertension, reductions in renal mass and/or a substantially reduced number of functional nephrons. Finally, we note that this model is almost always implemented in rats. PAN does not typically produce glomerular disease in wild-type mice but can do so if additional manipulations that compromise podocytes are simultaneously employed.

**Figure 2:**
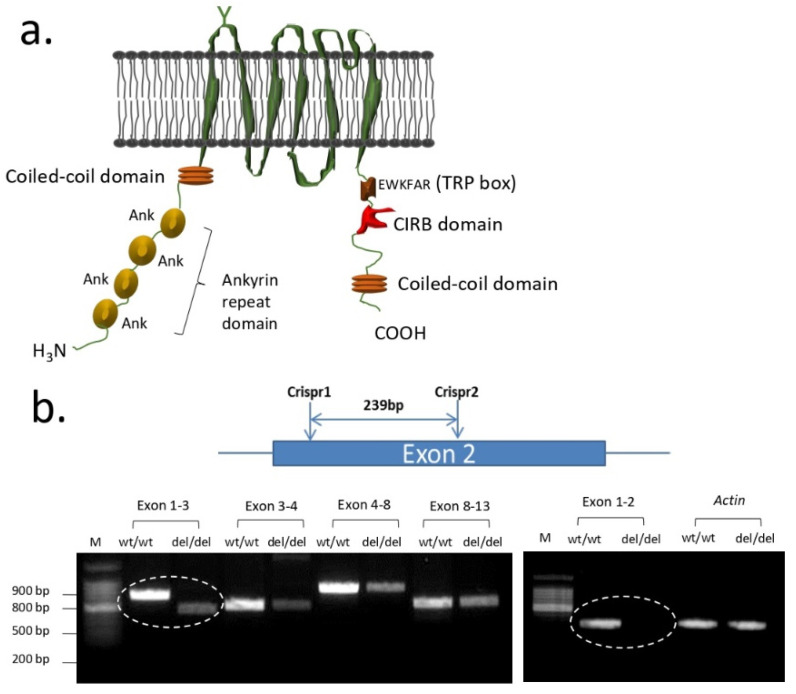
TRPC6 channel subunits and Crispr/Cas9 editing to create *Trpc6*^del/del^/rats. (**a**) Organization of TRPC6 subunits including ankyrin repeats on the amino-terminal cytosolic domain, the six transmembrane alpha-helices, and regulatory domains on the carboxyl-terminal cytosolic domain. (**b**) Crispr/Cas9 editing of the Trpc6 gene was used to delete a 239 bp region located in exon 2, which normally encodes ankyrin repeats 1 and 2. As a result of the deletion, the animals splice out all of Exon 2 in a post-translational process. RT-PCR analysis using primers designed to span the exons indicated shows that exon 2 is missing from the *Trpc6* transcripts isolated from kidneys of *Trpc6*^del/del^ rats but is present in their *Trpc6*^wt/wt^ littermates. More details are found in [50]. The proteins encoded by *Trpc6*^del^ do not form functional channels [50].

**Figure 3:**
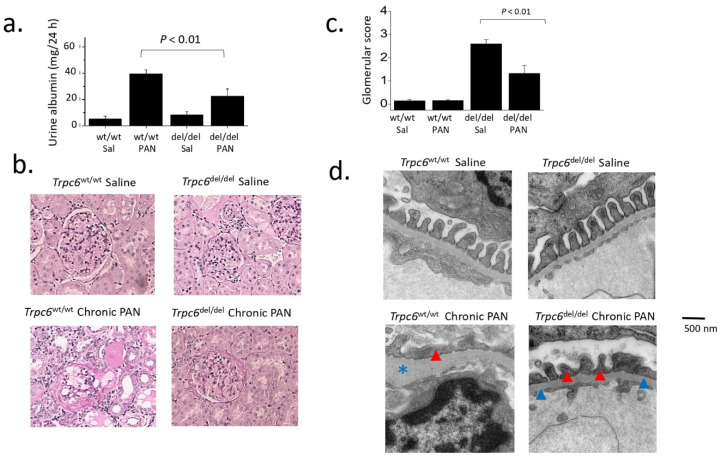
Effects of TRPC6 inactivation in the chronic PAN nephrosis model of adaptive FSGS. (**a**) *Trpc6*^del/del^ rats have reduced albuminuria during the chronic phase of PAN nephrosis compared to their *Trpc6*^wt/wt^ littermates. Urine was analyzed 60 days after the initial PAN injection. (**b**) Representative sections of renal cortex stained with periodic acid-Schiff’s show that glomerulosclerosis was less severe a *Trpc6*^del/del^ ras than in a *Trpc6*^wt/wt^ littermate. (**c**) Semiquantitative analysis of glomerulosclerosis based on experiments similar to that shown in (**b**), carried out on groups of animals. (**d**) Electron microscopy showing reduction in podocyte foot process effacement during chronic PAN nephrosis in a *Trpc6*^del/del^ rat compared to a *Trpc6*^wt/wt^ littermate. Foot processes are marked by red arrow heads. Note also markedly increased thickening of glomerular basement membrane (GBM) in micrograph from a *Trpc6*^del/del^ rat, marked by blue asterisk. Compare to normal glomerular basement membrane indicated by blue triangle. Additional details and analyses are in [50].

PAN nephrosis during both the acute and chronic phases has been examined in Sprague Dawley rats in which TRPC6 channels were globally inactivated using CRISP/Cas9 gene editing methods [50] (Figure 2). In those animals, a 239-bp region within exon 2 of *Trpc6* was deleted (thereby creating the *Trpc6*^del^ allele). Homozygous *Trpc6*^del/del^ animals express very small amounts of a truncated TRPC6 protein in which all of exon 2 is removed by a process of posttranscriptional skipping (Figure 2b). However, the resulting TRPC6^del^ subunits cannot assemble to form functional channels and therefore *Trpc6*^del/del^ animals function as global and constitutive knockouts [50]. For simplicity we will refer to them as knockouts hereafter. Wild-type littermates (*Trpc6*^wt/wt^ rats) were used as controls. Both types of animals exhibited high levels of albuminuria during the acute phase of the PAN model, measured 10 days after the initial PAN injection, and there was no difference in urine albumin excretion between *Trpc6*^wt/wt^ and *Trpc6*^del/del^ rats at that time point. A second PAN injection was given at 30 days, and animals were euthanized 30 days after that (i.e., 60 days after the first PAN injection). Both groups of animals exhibited albuminuria immediately prior to euthanasia. However, the 24-h urine albumin excretion in *Trpc6*^del/del^ animals was less than half that detected in the *Trpc6*^wt/wt^ animals, whereas saline treated animals had normal urine albumin excretion regardless of genotype [50] (Figure 3). In addition, *Trpc6*^del/del^ animals exhibited substantially less glomerulosclerosis, foot process effacement, or tubulointerstitial disease (Figure 3). They also exhibited marked improvements in serum creatinine concentration, blood urea nitrogen, glomerular expression of α-smooth muscle actin, or glomerular infiltration of CD68-expressing immune cells. TRPC6 knockout did not affect mean arterial pressure in Sprague Dawley rats [50]. Thus, constitutive and global TRPC6 knockout markedly reduces disease severity in an animal model of adaptive FSGS and by most indices disease severity was reduced by approximately 50%. By contrast, TRPC6 knockout did not produce detectable protective effects during the acute phase, a time when markedly increased ROS production is adversely affecting a host of molecular targets in podocytes [50]. TRPC6 knockout in the chronic PAN model also substantially reduced the severity of tubulointerstitial disease based on histological analysis and measurements of biochemical markers of inflammation and fibrosis [50]. It should be noted that the tubulointerstitial disease in the chronic PAN model occurs secondary to the glomerulosclerosis, in some cases because severely sclerotic glomeruli become detached from their tubules, and because of an accumulation of fibroblasts surrounding the sclerotic glomeruli that adversely affect adjacent tubular elements [71]. PAN also evokes marked albuminuria and glomerulosclerosis in FVB/NJ mice selectively expressing a mutant constitutively active form of the heterotrimeric G protein Gq in podocytes [59]. Moreover, when those animals were crossed with TRPC6 knockout mice the resulting albuminuria and glomerulosclerosis significantly was reduced, consistent with the conclusions of the studies carried out in rats. We will see further below that protective effects of TRPC6 knockout on tubulointerstitial compartments are not seen in every disease model but can be quite significant in some.

### 5.2. Anti-GBM Autoimmune Glomerulonephritis

**Figure 4:**
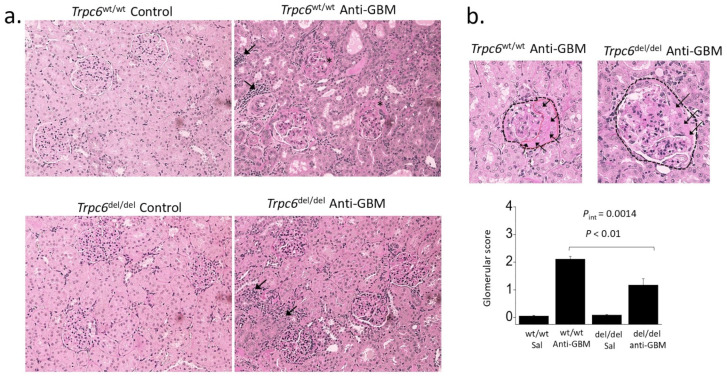
Glomerular damage and interstitial disease in anti-GBM glomerulonephritis in rats. (**a**) Note severe glomerulosclerosis in a *Trpc6*^wt/wt^ rat during autologous phase of anti-GBM nephritis. There is also marked hypercellularity in tubulointerstitial areas (arrows) and many of the tubules are dilated, and hyalinization is present. Glomerulosclerosis is present but is less severe in a *Trpc6*^del/del^ rat but tubulointerstitial disease is still present. (**b**) Semi-quantitative analysis shows that glomerulosclerosis is less severe during anti-GBM nephritis in *Trpc6*^del/del^ rats compared to *Trpc6*^wt/wt^ littermates. Additional details and analyses are in [51].

These same *Trpc6*^del/del^ rats and their *Trpc6*^wt/wt^ littermates have been examined in a model of autoimmune anti-glomerular basement membrane (GBM) glomerulonephritis [51]. The disease in these experiments was induced by intravenous injection of a sheep anti-rat GBM serum. The sheep antibodies recognize antigens in the GBM, which results in albuminuria that can be detected 4 days after the injection, i.e., during the heterologous phase of the immune response [69]. The proteinuria remains severe at 28 days after the injection by which time the rats have mounted an immune response to the sheep antibodies that have adhered to the GBM (the autologous phase of the response). At that time, it is possible to detect extensive depositions of rat IgG and complement within glomeruli, and serum creatinine and blood urea nitrogen are elevated, indicating a reduction in overall renal function. Urine markers of proximal tubule damage are also elevated, and there is extensive fibrosis and necrosis in tubules and in the interstitium that can be detected using a variety of different histochemical and biochemical markers. In addition, there are marked increases in TRPC6 and TRPC3 abundance in the glomeruli of *Trpc6*^wt/wt^ rats treated with anti-GBM serum compared to those treated with a control serum. TRPC6 knockout reduced glomerulosclerosis in this disease model as ascertained by semi-quantitative analysis of light microscopic images (Figure 4). By contrast, TRPC6 knockout did not reduce overall proteinuria, and did not produce any detectable protective effects in tubules or in the cortical or medullary interstitium [51]. This pattern is distinctly different from that seen in the chronic PAN model. It is possible that during anti-GBM glomerulonephritis, especially during the autologous phase, various fibrotic and inflammatory processes will occur in tubules and interstitium independent of the processes that initially lead to glomerular scarring. This may occur because immune cells activated within glomeruli subsequently move through the efferent arteriole and into other renal compartments [72,73] where they are able to drive inflammation and fibrosis.

An observation made by chance during these experiments is that cells robustly expressing the podocyte marker WT-1 can be found far outside of glomeruli in TRPC6 knockout rats subjected to anti-GBM glomerulonephritis. The mechanisms of this unusual effect are not known, but it is possible that these misplaced WT-1+ cells are derived from parietal cells that begin to acquire a podocyte phenotype following glomerular damage [74] but that the lack of functional TRPC6 channels causes them to migrate aberrantly. Additional studies are needed to understand this phenomenon.

### 5.3. Aging

Progressive declines in glomerular filtration rate (GFR) and renal blood flow (RBF) are a common feature of aging and at least some degree of glomerulosclerosis has been detected in approximately 70% of people over 40 years of age, with an average decline of nearly 10% of GFR and RBF occurring with each decade of life after the age of 40 [75]. Aging is associated with glomerular enlargement, widening of the GBM, mesangial expansion, and podocytopenia [76]. These changes are usually accompanied by increases in urine protein and albumin excretion [76,77]. In rats, these changes are accompanied by tubulointerstitial fibrosis [78]. It should be noted that the severity of age-related changes in renal structure and function depend on multiple factors including genetic background and diet [79].

TRPC6 knockout ameliorates some but not all the changes that occur during renal senescence in rats. Thus, *Trpc6*^del/del^ Sprague Dawley rats exhibited significantly less glomerulosclerosis than their *Trpc6*^wt/wt^ littermates at 12 months of age or beyond [52]. Despite that, *Trpc6*^del/del^ rats and their wildtype *Trpc6*^wt/wt^ littermates showed similar declines in renal function as assessed by blood urea nitrogen, the urine excretion of albumin and β2-microglobulin (the latter a marker of proximal tubule integrity), or the severity of tubulointerstitial fibrosis [52]. Thus, in rats the effect of TRPC6 knockout in aging is partially glomeruloprotective but this is not sufficient to preserve several of the most important aspects of renal function that decline during aging.

### 5.4. Diabetic Nephropathy

Declines in renal function occur in as many as 40% of patients with diabetes mellitus and is associated with a wide spectrum of changes in renal structure and function. Indeed, diabetes is the most common cause of ESKF in the world. Hyperglycemia is the hallmark feature of diabetes regardless of whether one considers type 1 or type 2 diabetes. While diabetes produces effects throughout the kidney, the earliest histological manifestations of diabetic nephropathy are seen in glomeruli, especially with respect to secretion of mesangial matrix and in the ultrastructure of podocyte foot processes. Hyperfiltration is usually seen very early during diabetes [80] even before the onset of microalbuminuria, and the accompanying increase in glomerular capillary pressure would be expected to cause an increase in TRPC6 activation in podocytes [25]. There is also a substantial literature indicating that elevated extracellular glucose increases the activity of podocyte TRPC6 channels secondary to increased generation of ROS [81] and as a result of increased signaling by Ang II [82]. Indeed, increased glomerular TRPC6 abundance is observed in diabetic mice and rats. Based on these observations it was predicted that TRPC6 knockout or inhibition would ameliorate diabetic nephropathy (DN). Therefore, it has been somewhat surprising that TRPC6 knockout by itself does not produce sustained renoprotection in rodent models of diabetes. The first detailed study was carried out in *Akita* mice, a genetic model of type 1 diabetes [60]. These animals were crossed with a line of *Trpc6* knockout mice that had been created many years earlier. In the *Akita* model, TRPC6 knockout was associated with reduced albuminuria in young animals (12–16 weeks of age). However, this protective effect was no longer observed once the animals reached 20 weeks of age. TRPC6 knockout also promoted resistance to insulin signaling in glomeruli and in isolated podocytes due to reduced expression of insulin receptor substrate 2 and an increase in the expression of cyclooxygenase 2 [60]. We previously noted that TRPC6 is not the only TRPC channel expressed in glomeruli, and it has been reported that a combined global knockout of TRPC3, TRPC6, and TRPC7 in mice reduced albuminuria and histological kidney damage in the streptozotocin (STZ) model of type 1 diabetes in mice up to at least 12 weeks of age [83], but it is not known if the protection by these multiple knockouts would have been more sustained, and it is not known if this strain of mice becomes insulin-resistant.

One limitation of studies of diabetic nephropathy in mice is that the renal disease in this species tends to be relatively mild, certainly compared to what occurs in humans. It is also highly dependent on genetic background. However, the effects of TRPC6 knockout have also been studied in diabetic rats, which typically show more severe renal disease. Thus, type 1 diabetes induced by STZ is associated with increased glomerular TRPC6 expression in Dahl SS rats. TRPC6 inactivation using CRISPR/Cas9 in that strain did not ameliorate elevated blood glucose or urine albumin excretion. There was however a small reduction in urine nephrin excretion, suggesting that the knockout decreased podocyte detachment and there was also some reduction in basal Ca^2+^ levels [61]. A similar result was obtained in Sprague Dawley rats, in which TRPC6 knockout produced no discernible protective effect on hyperglycemia, albuminuria, histological kidney injury, blood urea nitrogen, or plasma creatinine [62]. In that strain there was no decrease in urine nephrin excretion [62]. Moreover, in contrast to what was observed in mice, there were no indications of a protective effect even in young rats [62]. The differences in these various studies may reflect both species and genetic background, which can have significant effects on the severity of renal complications in diabetes [84]. The results of these studies have however suggested related therapeutic approaches. Thus, ROS generated by NADPH oxidase-4 leads to mobilization of TRPC6 in podocytes [85] and NOX4 knockout is protective in STZ-induced diabetes and is associated with markedly reduced Ca^2+^ influx in podocytes from diabetic Dahl SS rats [86]. GLP-1 agonists, such as liraglutide, reduce the expression of glomerular TRPC6 in STZ-induced diabetes in Sprague Dawley rats and may act to reduce interactions between TRPC6 and NADPH oxidases [87]. Thus, it is possible inhibition of TRPC6 may not be sufficient to ameliorate diabetic nephropathy but might potentiate effects of other therapeutic strategies.

### 5.5. Tubulointerstitial Fibrosis and Acute Kidney Injuries

**Figure 5:**
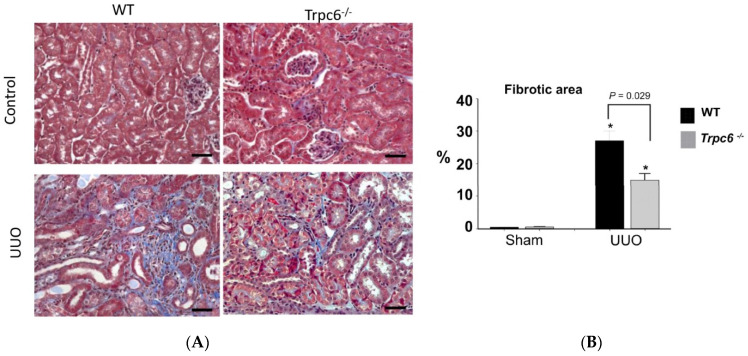
Reduction in renal fibrosis in Trpc6^−/−^ mice following unilateral ureteral obstruction. (**A**) Histological analysis based on Masson’s trichrome stain. (**B**) Quantitative analysis of this type of experiment from many animals. Asterisks indicate *p* < 0.05 compared to sham. Modified from [64] and used with permission.

All the models described above exhibit substantial glomerular involvement at the earliest stages of the disease. However, TRPC6 channels have also been implicated in disease processes in which the initial pathology is observed in distal tubules and in the interstitium and in which there is minimal glomerular involvement. This has been most extensively studied in the unilateral ureteral obstruction (UUO) model in mice [64,65,66]. UUO induces a kidney injury that occurs initially within distal tubular epithelia, but which has sequelae that affect other parts of the kidney. UUO induces tubular dilation, interstitial expansion, loss of proximal tubular mass, hydronephrosis, infiltration of leukocytes and activated fibroblasts, and tubuloepithelial cell death. The fibrosis that occurs after UUO in mice occurs with a rapid onset compared to that which occurs in humans following obstruction of the urinary tract [88]. Several studies have now reported reductions in tubulointerstitial fibrosis in TRPC6 knockout mice subjected to UUO, based on extensive histological analysis and measurement of numerous biochemical and circulating markers [64,65,66]. A representative example from the first of those studies is shown in Figure 5. These investigators also found that administration of soluble klotho evoked a decrease in TRPC6 expression throughout the kidney and reduced UUO-induced tubulointerstitial fibrosis in wild-type but not in TRPC6 knockout mice [64]. These conclusions are supported by pharmacological data. Thus, the selective TRPC6 inhibitor BI 749327 [89], and the pan-TRPC inhibitor 4-methyl-4-[3,5-bis(trifluoromethyl)-1H-pyrazol-1-yl]-1,2,3-thiadiazole-5-carbox anilide (BTP2) [64] have been observed to reduce renal fibrosis induced by UUO [64,89] well as cardiac fibrosis and hypertrophy in mice subjected to chronic pressure overload [89]. In addition, a structurally distinct TRPC6 inhibitor known as SH045 reduces renal fibrosis in an obesity model of metabolic syndrome [90]. Thus, TRPC6 knockout or inhibition in mice appears to have a non-trivial anti-fibrotic effect in at least two different tissues.

UUO is a model of a post-renal obstructive injury, but many cases of AKI are a consequence of pre-renal mechanisms in which there is extensive ischemia. The results published to date on the role of TRPC6 in ischemia-reperfusion models of AKI have reached different conclusions. Thus, in mice on a mixed 129 Sv:C57BL/6J background, TRPC6 knockout had no effect on the expression of epithelial damage markers, or on the tubular injury or the inflammatory response assessed by histological analyses [67]. Moreover, TRPC6 abundance was not increased by this manipulation, and administration of the TRPC6 inhibitors SH045 or BI-749327 to C57NL/6J mice had no effect on ischemic AKI in this model [67]. By contrast, 129CvEv mice subjected to ischemia-reperfusion exhibited a marked increase in renal TRPC6 abundance and TRPC6 knockout resulted in a significant reduction in renal injury as assessed by both histology and measurement of biochemical markers of inflammation, autophagy, apoptosis, and mitochondrial function [68]. A similar result was obtained in cultured tubular epithelial cells [68]. The reasons why these studies reached different conclusions are not known, but it bears noting that the duration of the renal ischemia was different in the two studies (20 min in [67] vs. 40 min in [68]) and the experiments were carried out on mice from different genetic backgrounds.

TRPC6 knockout has also been examined in the albumin overload (AO) model of renal fibrosis in rats [63]. In this model, rats are administered a heterologous form of exogenous albumin, usually bovine serum albumin, by daily intraperitoneal injections over a period of 2–6 weeks. This protocol produces hyperalbuminemia and albuminuria that is accompanied by damage to proximal tubules, tubulointerstitial nephritis and fibrosis [91]. There is also mild pathology in glomeruli, for example, there is an increase in phagocytic granules within podocytes. This is not an autoimmune model, rather it is generally thought that proximal tubule cells are damaged following endocytosis and lysosomal processing of excessive quantities of albumin [91]. Proximal tubule cells secrete a variety of factors in response to protein overload that can contribute to the overall tubulointerstitial injury [92], although it is possible that glomerular cells contribute to this process [93]. We recently observed that AO in Sprague Dawley rats resulted in an increase in the abundance of TRPC6 and TRPC3, which, surprisingly, was accompanied by a nearly complete loss of TRPC5 in the renal cortex [63]. Moreover, TRPC6 knockout rats (*Trpc6*^del/del^) exhibited a 40–50% reduction in albuminuria following 2 or 4 weeks of AO compared to wildtype littermate (*Trpc6*^wt/wt^) controls [63]. Similarly, knockout of syndecan-4, which results in marked reduction of glomerular TRPC6 expression in mice, also reduced urine albumin excretion following AO [94]. Despite the reduction in proteinuria, there was no reduction in tubulointerstitial disease in rats seen with TRPC6 knockout [63]. It is possible that the reduction in urine albumin in the TRPC6 knockout animals was insufficient to prevent proximal tubule cells from triggering pro-fibrotic and pro-inflammatory signals. It is also possible that some of the pro-inflammatory signals that trigger tubulointerstitial disease during AO originate in glomeruli and are unaffected or enhanced by TRPC6 knockout.

## 6. Implications and Limitations of Studies on TRPC6 Knockout Animals for Drug Development

Studies using TRPC6 knockout animals support continuing drug discovery efforts that target TRPC6 channels directly or indirectly for therapy of kidney diseases. From a purely biological perspective, the strongest case for this class of drugs would be for people with familial nephrotic syndromes that are caused by one of the gain-of-function mutations of TRPC6, and it bears noting that SH045, which is a congener of the natural product (+)-larixol, is able to block those mutant TRPC6 variants [90]. Agents that block or suppress TRPC6 may also be effective in other genetic forms of FSGS, such as those that are associated with mutations in *NPHS2* [23]. Based on the actions of putative serum “permeability factors” on TRPC6 channels in podocytes, including samples taken from patients, it is possible that TRPC6 inhibitors could be efficacious in people with primary FSGS [53,54], and knockout studies in rats support the concept of TRPC6 inhibition in adaptive forms of FSGS [50], commonly seen in patients with severe uncontrolled hypertension, reduced renal mass, or a markedly reduced number of functional nephrons. Based on studies in knockout mice, there is evidence that TRPC6 inhibition may be useful to reduce tubulointerstitial fibrosis, especially if it is caused by urinary tract obstruction [64,65,66], and, importantly, these conclusions are supported by pharmacological data [64,89,90]. Finally, and somewhat unexpectedly, the effects of TRPC6 knockout in diabetic kidney disease models in rodents have been disappointing [60,61,62].

A limitation of nearly all preclinical studies is the issue of species differences and difference due to genetic background. Mice and rats are not people and ion channels may have somewhat different functions in various tissues depending on species. In addition, the disease models used in rats and mice are not perfect copies of what occurs in humans. For example, diabetic kidney disease in rats and especially in mice is not as severe as it is in humans, shows somewhat different histological characteristics, and is highly dependent on genetic background [84]. In addition, there are no widely available animal models for relatively common kidney diseases in humans, such as primary FSGS. Other models, such as acute PAN nephrosis and the use of protamine sulfate, are not really models for any human disease, although they certainly damage glomeruli and may yield useful insights. On the other hand, autoimmune, ischemia-reperfusion, and urinary tract obstruction models recapitulate many aspects of common human kidney disorders and injuries [69,88]. Finally, one needs to consider the issue of lifespan and how it affects the development of a kidney disease. A human with a TRPC6 mutation may experience renal failure in the third decade of life [35], which would be considered quite young relative to a normal human lifespan, and yet an adult mouse constitutively over-expressing the same TRPC6 variant may barely show any effects [46,47]. Perhaps this reflects the fact that laboratory mice only live for 24–30 months, or perhaps it reflects the fact that mice have a great deal of spare capacity in their kidneys.

All of the TRPC6 knockout models published to date have been constitutive and global, which raises the possibility of effects on pre- or post-natal developmental processes. It also raises the possibility of compensatory changes in the expression of other genes. For example, at least at the level of protein abundance, TRPC6 knockout rats have an increase in TRPC3 expression in the kidneys, although at least in podocytes the TRPC3 channels are not sufficient to sustain signaling by themselves [19,50]. That is almost certainly not true in every cell type or tissue. Indeed, compensatory increases in TRPC3 can lead to an increase in contractility of vascular smooth muscle and hypertension in TRPC6 knockout mice [95], although the extent to which this occurs depends on genetic background [96]. Compensatory changes would be unlikely to occur to the same extent with pharmacological inhibition of TRPC6, which would normally be less complete, initiated later in life, and subjected to fluctuations related to the pharmacokinetics of a drug. In this regard, it might be useful to study agents that inhibit TRPC6 and TRPC3, many of which have been discovered. For example, BTP2 was efficacious in the UUO model of renal fibrosis [64]. Finally, we note that TRPC6 knockout is a model of a monotherapy. However current therapy of kidney disease almost always entails the use of multiple agents, and it is possible that TRPC6 inhibition would be more effective if other targets were simultaneously blocked, especially in the case of diabetic nephropathy or hypertension.

In summary, a strong argument can be made for continued drug discovery efforts focused on agents that modulate directly or indirectly the activity of TRPC6 channels and related members of the TRP family of cationic channels.

## Figures and Tables

**Table 1:** Summary of effects of TRPC6 knockout in animal models of kidney diseases.

Model	Species	Result	Ref.
Acute PAN nephrosis	Rat (Sprague Dawley)	No protective effect on urine albumin excretion	[50]
Chronic PAN nephrosis	Rat (Sprague Dawley)	Reduced glomerulosclerosis, albuminuria, reduced tubulointerstitial disease, improved overall renal function	[50]
Acute PAN nephrosis in mice over-expressing constitutively active G_q_ in podocytes	Mice (FVB/NJ)	Reduced albuminuria and glomerulosclerosis and improved overall renal function	[59]
Autoimmune anti-GBM glomerulonephritis	Rat (Sprague Dawley)	Reduced glomerulosclerosis but no effect on interstitial fibrosis	[51]
Aging	Rat (Sprague Dawley)	Reduced glomerulosclerosis but no effect on decline in overall renal function	[52]
Diabetic nephropathy (Akita mouse model)	Mice (*Akita* FVB/NJ)	Transient protection in young animals but later insulin resistance occurs by 20 weeks of age along with loss of renal protective effect	[60]
Diabetic nephropathy (STZ model)	Rat (Dahl SS)	No effect on histology or urine albumin excretion but reduced urine nephrin excretion	[61]
Diabetic nephropathy (STZ model)	Rat (Sprague Dawley)	No effect on histology, urine albumin excretion or urine nephrin excretion	[62]
Albumin overload	Rat (Sprague Dawley)	Reduced urine albumin excretion but no effect on interstitial fibrosis	[63]
UUO	Mice (129/SvJ)	Reduced interstitial fibrosis	[64]
UUO	Mice (C57BL/6J)	Reduced interstitial fibrosis	[65]
UUO	Mice (129/SvEv)	Reduced interstitial fibrosis and inhibition of epithelial-mesenchymal transition	[66]
Ischemia-reperfusion	Mice (mixed 129/Sv: C57BL/6J)	No protective effect	[67]
Ischemia-reperfusion	Mice (129/SvEv)	Reduced tubular damage based on histology and biochemical markers	[68]
